# A Rare Case of an Esophageal-Pericardial Fistula With Large Pericardial Effusion and Pyopneumopericardium in the Setting of Gastric Adenocarcinoma

**DOI:** 10.7759/cureus.84199

**Published:** 2025-05-15

**Authors:** Samer Farhud, Michael Anzelmo, Najat Y AlSejari

**Affiliations:** 1 Internal Medicine, Ochsner Medical Center, New Orleans, USA; 2 Internal Medicine, Jaber Hospital, Kuwait City, KWT

**Keywords:** esophageal-pericardial fistula, gastric adenocarcinoma, gastric cancer, palliative medicine, pericardial effusion, pyopneumopericardium, tamponade, tamponade pneumopericardium

## Abstract

An esophageal-pericardial fistula (EPF) is a rare, life-threatening complication of esophageal disease, most commonly occurring due to esophageal carcinoma. It can result in pericardial effusion, cardiac tamponade, and air in the pericardium or cardiac chambers, further complicating the clinical course. An EPF usually occurs in esophageal-related cancers and, less commonly, gastric-related cancers. This study presents a case of an EPF in an 86-year-old male with adenocarcinoma of the gastric cardia, complicated by a large pericardial effusion with transient tamponade physiology. Imaging, such as a CT esophagram, confirmed tract communication, and supportive imaging, such as echocardiograms, confirmed complications.

## Introduction

An esophageal-pericardial fistula (EPF) is a rare but life-threatening complication of esophageal disease that occurs secondary to disorders such as chronic esophagitis, hiatal hernia, malignant esophageal carcinoma, iatrogenic injury, foreign body impaction, and congenital anomalies [1]. It is accompanied by a high mortality rate, especially in cases where the fistula is large or remains undiagnosed earlier in its course of development [2-3]. The incidence number of these fistulas in gastric adenocarcinoma is unaccounted for, as this is a rare complication as opposed to fistula in esophageal pathologies. The literature reports an incidence of 24% of known esophageal fistulas, being secondary to malignancy. This unfortunately does not specify the type of fistula or type of malignancy. If untreated, an EPF can lead to severe complications including cardiac tamponade, pericarditis, pneumo-pericardium, pyopneumo-pericardium, and sepsis [4-5]. Thus, rapid diagnosis and treatment are crucial to avoid permanent disability and death. 

Pericardial effusions are characterized by the accumulation of fluid within the pericardial sac that is greater than the physiological range between 15 and 50 mL [5]. The composition of the fluid can range from the possible causes of the underlying effusion. If there is pus accumulation, this would be classified as a pyopericardium; air would indicate a pneumopericardium, blood would indicate a hemopericardium, and chyle would indicate a chylopericardium. The etiology of the underlying fluid collection can be from a range of conditions, such as autoimmune (rheumatoid or lupus), Ischemic heart disease, malignancies, and infections [5]. 

It is most accurately diagnosed via chest CT with oral contrast, which can identify the fistulous tract and its associated complications [6]. Other imaging modalities, such as chest X-rays and echocardiography, can provide supportive findings but not identify the tract. Treatment depends on the severity of the condition; however, it generally relies on pericardial drainage, antibiotic therapy, esophageal stenting, and, in advanced cases, surgical repair [7]. 

In this case report, we present a rare case of an 86-year-old gentleman with adenocarcinoma of the gastric cardia who was found to have an EPF and pneumopericardium, which was found with CT esophagram with oral contrast. Pericardial fluid culture was positive for *Candida glabrata*, indicating a complication of pyopneumopericardium, which required antifungal treatment.

## Case presentation

An 86-year-old male, with a history of metastatic malignant neoplasm of the cardia of the stomach (actively receiving chemotherapy), esophageal stent placement (due to tumor burden), deep vein thrombosis (on apixaban), chronic kidney disease (stage 3), hypertension, and diastolic heart failure, presented at an outside facility with complaints of chest and abdominal pain for several days. He denied nausea, vomiting, fever, cough, or changes in urinary or stool habits. En route to the hospital, the patient was found to be hypotensive with a blood pressure of 60/40 mmHg. Emergency medical services (EMS) administered 200 ml of normal saline. Upon arrival at the facility, his blood pressure (BP) was noted to be 98/46 mmHg, and the patient was afebrile. During his emergency department (ED) stay, a CTA chest with contrast was performed, which noted large pericardial effusion with pericardial enhancement, with air also noted in the pericardial sac. Due to the need for surgical consultation, the patient was transferred to a hospital with cardiothoracic services for further evaluation. 

Upon arrival at the hospital, his blood pressure was 94/55 mmHg, heart rate was 79 beats per minute, respiratory rate was 20 breaths per minute, temperature was 36.4°C (97.5 °F), and hemoglobin was 7.2 g/dL (near baseline of 7-8). 

**Figure 1:**
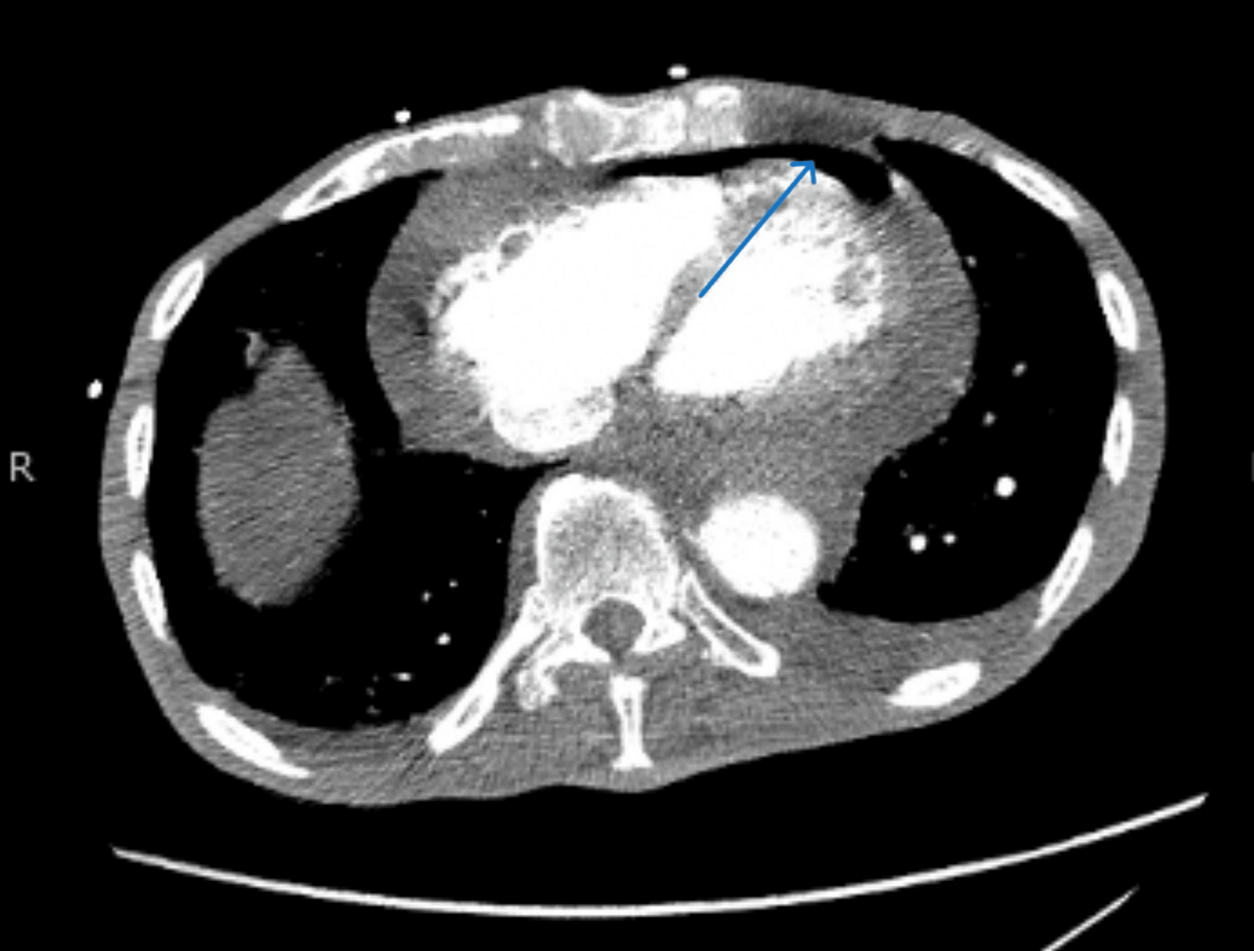
Large pericardial effusion with evidence of free air in the pericardial sac as evident by the blue arrow

His laboratory results are displayed in Table 1. The patient presented with normal urinalysis in addition to these lab values. Imaging done at an outside facility consisting of CTA chest and CT abdomen/pelvis with contrast revealed the interval development of a large pericardial effusion with pericardial enhancement and the presence of air within the pericardial sac (Figure 1). In addition, air was noted outside of the posterior segment of the left atrium abutting the esophagus, and there was thickening of the distal esophagus extending into the periaortic region, suggesting worsening malignancy. No evidence of dissection or pulmonary embolism was noted, and the esophageal stent remained in place.

**Table 1:** Patient lab values upon admission WBC: white blood cell, INR: international normalized ratio

Lab Value		Reference value
WBC	10 x 10^9^ cells per liter	4.5-11^9^ cells per liter
INR	1.1	0.8-1.1
D-dimer	16.58 fibrinoegen equivalent units	<0.50 fibrinogen equivalent units
Creatinine	1.1 mg/dL	0.7-1.3 mg/dL
Lactate	3.3 mmol/L	0.5-2.2 mmol/L
Potassium (K)	3.3 mmol/L	3.5-5.0 mmol/L

Based on these findings, broad-spectrum antibiotics were initiated, and cardiothoracic surgery recommended further imaging. A CT esophagram confirmed the diagnosis of a PEF. After discussions with the cardiothoracic surgery team, the patient consented to esophageal stent placement and a pericardial window. In the ICU, more than 1 liter of serosanguinous pericardial fluid was drained from the pericardium. An esophagram done after placement of the esophageal stent showed no evidence of persistent communication between the esophagus and pericardium. The pericardial effusion was cultured and came back positive for *Candida glabrata*, and he was subsequently transitioned from piperacillin/tazobactam to micafungin for the management of the pyopneumopericardium with a planned course of four weeks per infectious disease recommendations. Due to the patient’s complex clinical course, palliative care was consulted to assist in further management. He was ultimately discharged with plans to complete the antifungal course and transition to home with hospice care once the family and patient felt ready.

## Discussion

An EPF is an unusual but disastrous complication of esophageal and, less commonly, gastric malignancies. It is an abnormal connection between the esophagus and the pericardium through which food, air, fluid, and infective organisms gain access to the pericardial space [1]. Presentation can be nonspecific and may range from chest pain, dyspnea, dysphagia to features of sepsis or pericardial tamponade, making early diagnosis challenging. As illustrated by our case, an EPF in the setting of advanced adenocarcinoma of the stomach is rare, and few cases are described in the literature. Epiphrenic foramen enlargement is more typically associated with esophageal cancer and most frequently with locally advanced disease with direct invasion into adjacent structures [2]. Gastric adenocarcinomas, such as adenocarcinoma of the cardia, have been less commonly implicated in this complication, but the current case sheds light on the possibility of such an association. 

Mortality for an EPF remains high, especially in the setting of delayed diagnosis or absence of early intervention [3]. The fistula can cause life-threatening complications such as pericardial effusion, cardiac tamponade, pneumopericardium, pyopneumopericardium, and sepsis. In this case, the discovery of a large pericardial effusion with air in the pericardial sac and posterior to the left atrium indicated not only the mechanical complications of an EPF but also the infectious aspect of the disease [3-5]. The infection of the pericardial effusion in our patient was particularly troubling. Effusion cultures grew *Candida glabrata*, implying a concomitant pyopneumopericardium. This is a reminder of the risk of fungal infection in EPF patients, particularly those with underlying malignancy or immunocompromised states. 

Candida glabrata is an opportunistic pathogen that typically infects immunocompromised patients, such as those undergoing chemotherapy or with chronic illnesses [5-6]. The presence of this pathogen in the pericardial fluid complicated the patient's management and necessitated the use of antifungal therapy. The patient was initially started on empiric antibiotics with a transition to antifungals once culture data resulted, indicating the rarity of pericardial fungal infections. Antifungal therapy in the management of pyopneumopericardium complicating an EPF is crucial in order to limit further systemic spread of infection and to improve patient outcomes. Diagnosis and intervention in an EPF at an early stage are decisive for an improved prognosis for the patient. In this patient, further imaging studies, i.e., CT esophagram with oral contrast, were very important in identifying the fistula and its complications [7]. CT imaging is the gold standard for diagnosing an EPF because it provides near visualization of the pericardial effusion, air, and fistulous tract. Once diagnosed, treatment typically involves pericardial drainage to relieve tamponade and impending cardiovascular collapse [8]. Here, more than 1 liter of serosanguinous pericardial fluid was drained, which dramatically improved the patient's hemodynamic instability. The patient also underwent esophageal stenting to prevent any future communication between the esophagus and pericardium, a lifesaving maneuver in the fistula management. Despite these, the long-term outlook in patients with EPFs, especially those with very advanced malignancy, remains poor. In our patient, with the underlying gastric adenocarcinoma and widespread disease, palliative care was consulted for comfort and end-of-life concerns. The patient was discharged to hospice care in light of his grim prognosis and complex clinical course. This case illustrates the importance of early recognition and management of EPFs, particularly in patients with advanced cancer, to prevent fatal outcomes. 

## Conclusions

An EPF is a lethal and unusual complication that requires early diagnosis and management. The additional complexity of the pericardial space being infected, in this instance with *Candida glabrata*, adds yet another dimension of difficulty in the management of this illness. To our knowledge and literature review, this represents one of the few reported cases of EPFs with concomitant *Candida glabrata* infection, highlighting the rarity and diagnostic challenge of this presentation. The contribution of multidisciplinary management with cardiothoracic surgery, infectious disease consultation, and palliative care cannot be overstated in the management of such difficult cases. Early diagnosis, appropriate antimicrobial therapy, and prompt surgical intervention can greatly improve patient survival and quality of life in this challenging clinical scenario.
